# Comparison of Heart Rate Variability Between Sexes: Impact of a Physically Active Lifestyle

**DOI:** 10.3390/ijerph23060809

**Published:** 2026-06-18

**Authors:** Reberth Magalhães Da Silva, Ariane Viana, Fernanda Monma, Fernando Alves Santa Rosa, José Robertto Zaffalon, Kátia De Angelis

**Affiliations:** 1 Translational Physiology Laboratory, Universidade Nove de Julho (UNINOVE), Sao Paulo 01525-000, SP, Brazil; reberthmagalhaes@yahoo.com.br (R.M.D.S.); ari_viana@hotmail.com (A.V.); fmonma86@gmail.com (F.M.); fernandoasr78@yahoo.com.br (F.A.S.R.); jrzaffalon@uepa.br (J.R.Z.J.); 2 Military Police Physical Education School, Sao Paulo 03033-020, SP, Brazil; 3 Laboratory of Physical Exercise and Lifestyle (LEFEV), State of Para University (UEPA), Belém 66050-540, PA, Brazil; 4 Department of Physiology, Federal University of Sao Paulo (UNIFESP), Sao Paulo 04023-062, SP, Brazil

**Keywords:** heart rate variability, sex, physical activity, sedentary behavior

## Abstract

**Highlights:**

**Public health relevance—How does this work relate to a public health issue?**

- Physical inactivity, a major global public health concern, is associated with impaired HRV and increased cardiovascular risk.
- This study investigates the interaction between sex and physical activity on HRV, a marker of cardiovascular health.

**Public health significance—Why is this work of significance to public health?**

- The findings demonstrate that both female sex and a physically active lifestyle are associated with more favorable cardiac autonomic modulation, suggesting the contribution of both biological and behavioral factors to cardiovascular autonomic health.
- The present findings further support the relevance of HRV as a non-invasive tool for the early identification of alterations associated with cardiovascular risk in healthy populations.

**Public health implications—What are the key implications or messages for practitioners, policy makers and/or researchers in public health?**

- The promotion of regular physical activity may represent an important non-pharmacological approach associated with better cardiovascular autonomic regulation in both sexes.
- Public health policies and research should consider sex-specific physiological differences when designing prevention strategies and interventions targeting cardiovascular risk.

**Abstract:**

Sex differences and lifestyle factors such as physical activity play an important role in cardiovascular autonomic regulation. Heart rate variability (HRV) is a widely used marker of cardiac autonomic modulation and cardiovascular health. However, the combined influence of sex and physical activity levels on HRV in young, healthy adults has not been sufficiently explored. Therefore, this study investigated the effects of sex and a physically active lifestyle on HRV in men and women. A cross-sectional study was conducted on a cohort of young, healthy adults aged 18–30 and categorized into four groups based on: physically active men (AM; *n* = 37), sedentary men (SM; *n* = 44), and physically active women (AW; *n* = 31) and sedentary women (SW; *n* = 40). Regarding the impact of lifestyle, the AM group exhibited 41% higher total variance (VAR-RR) and 34% higher RMSSD (a parasympathetic index) than the SM group. The AW exhibited 74% and 78% higher VAR-RR and RMSSD, respectively, compared to the SW. Furthermore, the physically active groups (AM and AW) displayed a 75% and 50% lower LF/HF ratio, respectively, compared to their sedentary counterparts. Interestingly, the LF/HF ratio was 66% higher, and the RMSSD was 20% lower in the AM group than in the AW group. HRV indices demonstrated large to very large effect sizes. In conclusion, these findings demonstrate significantly advantage in cardiac autonomic modulation among physically active individuals and women. Together, these results reinforce the roles of female sex and an active lifestyle as important protective factors for cardiovascular health.

## 1. Introduction

Women are often underrepresented in studies exploring physiological and pathophysiological conditions, as well as in clinical trials evaluating pharmacological and non-pharmacological therapies [1,2]. However, research has indicated that men may experience a higher prevalence of cardiovascular disease (CVD) at an earlier age compared to pre-menopausal women, who appear to benefit from the antioxidant properties of estrogens [3,4]. It is worth noting that heart rate variability (HRV) is commonly used as a health indicator, with reduced HRV associated with increased cardiovascular risk and mortality [5]. In addition, autonomic imbalance characterized by reduced vagal modulation and increased sympathetic activity has been implicated in the pathophysiology of hypertension and may precede its clinical manifestation [6,7]. Previous studies have also highlighted sex-related differences in HRV parameters, with women generally displaying a lower resting heart rate, greater cardiac vagal tone, and lower cardiac sympathetic tone compared to men [8].

Moreover, the issue of physical inactivity has emerged as a significant and escalating public health concern affecting both sexes [9]. Globally, the prevalence of insufficient physical activity stands at 23.4% in men and approximately 31% in women [10]. Several studies have consistently shown that a sedentary lifestyle is closely linked to an elevated risk of CVD [11,12,13]. Furthermore, physical inactivity has been associated with a reduction in HRV [14]. Conversely, prior research has established that regular exercise training yields positive effects on cardiovascular autonomic control [5,15,16,17], even in individuals with hypertension and obesity [18,19]. However, although several studies have evaluated HRV separately in men or women, relatively few have directly compared both sexes, and the interaction between habitual physical activity levels and sex remains insufficiently elucidated, particularly in observational population-based studies.

In the context of our study, we hypothesized that sedentary women may exhibit higher vagal modulation compared to men, and that levels of physical activity may exert a sex-specific influence on these patterns. Therefore, our investigation sought to elucidate differences between sexes and the impact of physical inactivity on HRV in healthy men and women.

## 2. Methods

The observational, cross-sectional, and analytical study was conducted at the Laboratory of Translational Physiology of the University Nove de Julho (UNINOVE). Recruitment and data collection took place between September 2016 and June 2019. The dataset analyzed in the present manuscript was derived from participants recruited under three related protocols conducted by our research group and approved by the same Research Ethics Committee (CAAE: 46439315.9.0000.5511 date: 11 August 2016; CAAE: 49446315.5.0000.5511 date: 11 August 2016; CAAE: 83135918.9.0000.5511 date: 06 June 2018). Therefore, the final sample represents a combined dataset from these approved studies. This enabled comparison of sedentary and physically active men and women through assessment of anthropometric, metabolic, hemodynamic, and autonomic modulation parameters. All procedures conducted during the study were in strict compliance with the principles set forth in the 1975 Helsinki Declaration, revised in 2008. Furthermore, all participants were fully informed about the research procedures and provided informed consent via a formal consent form. 

A total of 152 male and female participants were recruited through convenience sampling. The participants were then categorized into four groups according to their responses to the International Physical Activity Questionnaire (IPAQ): active men (AM, *n* = 37), sedentary men (SM, *n* = 44), active women (AW, *n* = 31), and sedentary women (SW, *n* = 40).

Our study included young adults between 18 and 30 years old with a body mass index (BMI) between 18 and 24.99 kg/m^2^. Individuals who were smokers or taking medications that could potentially influence blood pressure levels were excluded from the study. These medications included thyroid hormones, non-steroidal anti-inflammatory drugs, antidepressants, illicit substances, and other medications.

Hemodynamic and autonomic responses were assessed during the follicular phase of the menstrual cycle or the high-hormonal phase in women using hormonal contraceptives, following the protocol as done by Zafallon et al. in 2018 [14]. Menstrual cycle phase was determined based on self-reported menstrual history and cycle timing, without biochemical hormonal confirmation. Prior to the evaluation day, all participants were instructed to abstain from consuming caffeine, chocolate, nicotine, alcohol, and other stimulating substances, as well as to avoid strenuous physical activity, for at least 24 h before the test. Measurements were taken under standardized resting conditions. Upon arriving at the clinic for the assessments, participants were instructed to remain seated for at least 15 min. These instructions were reinforced over the two days of assessments. Day 1: body composition assessment and IPAQ application. Day 2: hemodynamic assessments and HRV.

This work was developed following the recommendations in the STROBE Statement.

To assess physical activity levels and minimize potential measurement bias, trained researchers conducted standardized interviews using the International Physical Activity Questionnaire (IPAQ, version 6). Physical activity level was assessed using the IPAQ, and participants were classified according to its standard scoring criteria. Specifically, individuals were categorized as “insufficiently active” when they did not meet the minimum recommendations for moderate-to-vigorous physical activity (i.e., <600 MET-min/week), and as “sufficiently active” when they reached or exceeded this threshold (≥600 MET-min/week), as defined by the IPAQ scoring protocol. In the present study, participants classified as “sedentary” or “insufficiently active” were allocated to the sedentary groups (SM or SW), while those classified as “active” or “very active” were included in the active groups (AM or AW). Body composition was evaluated by the body mass index (BMI).

Resting heart rate (HR), systolic blood pressure (SBP), and diastolic blood pressure (DBP) were measured. Baseline SBP, DBP, and HR were measured three times after 15 min of sitting. Casual blood pressure was measured with an appropriately sized cuff and a mercury sphygmomanometer by the same researcher. The mean of the three readings was used for analysis [7].

HRV measurements were performed in a quiet, temperature-controlled room (approximately 23–24 °C) with the participants seated comfortably with their arms relaxed and their feet flat on the floor. This followed a standardized rest period of at least 15 min. HRV parameters were assessed by recording the RR interval with a Polar^®^ V800 heart rate monitor (Manufacturer name: Polar Electro Oy, Professorintie 5. FI-90440 KEMPELE.). The RR interval was recorded for 15 min while the subjects were at rest. The spectrum resulting from Fast Fourier Transform (FFT) modeling includes the entire signal variance, regardless of whether its frequency components appear as specific spectral peaks or as non-peak broadband power [20]. RR interval variability was evaluated in the time and frequency domains. RMSSD (root mean square of successive RR interval differences) reflects variability in NN interval changes. The spectral power in the low (LF: 0.04–0.15 Hz) and high (HF: 0.15–0.4 Hz) frequency bands, commonly interpreted as reflecting mixed autonomic influences and parasympathetic modulation, respectively, was calculated using a customized routine (Cardioseries) [14,20,21].

The results are presented as the mean ± standard error. Levene’s test was used to assess variance homogeneity. A two-way analysis of variance (ANOVA) was performed to compare the four groups, followed by a Bonferroni post hoc test using SPSS 22. The adopted significance level was *p* < 0.05. Sample power was calculated a posteriori, considering group variances for the RR interval and LF/HF ratio, with a β of 0.99 for both parameters (G*Power 3 software). Effect sizes were calculated using Cohen’s d for pairwise comparisons. Confidence intervals (95%) were estimated based on standard errors. Additionally, partial eta squared (η^2^) was derived from the two-way ANOVA model to estimate the magnitude of interaction effects. All analyses were conducted using procedures implemented in SPSS version 22.0 (IBM Corp., Armonk, NY, USA).

## 3. Results

**Figure 1:**
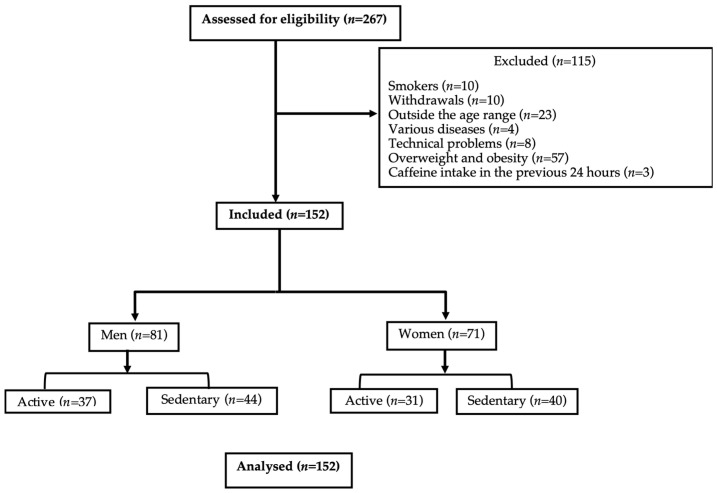
Study flowchart. STROBE Statement.

Our study included a final cohort of 152 volunteers who were divided into four distinct groups, as illustrated in Figure 1. Complete data were available for all assessed variables, with no missing values.

There were no significant differences in terms of age (AM: 24 ± 0.6 years; SM: 23 ± 0.5 years; AW: 23 ± 0.7 years; SW: 22 ± 0.6 years) or BMI across the groups (AM: 23 ± 0.3 kg/m^2^; SM: 22 ± 0.2 kg/m^2^; AW: 22 ± 0.5 kg/m^2^; SW: 22 ± 0.4 kg/m^2^). Moreover, effect size analyses demonstrated trivial differences for both variables (Cohen’s d < 0.20), supporting the baseline homogeneity of the study groups.

The hemodynamic parameters of the participants are presented in Table 1. Lower SBP values were observed in the AW and SW groups compared with the SM and AM groups, respectively. In addition, the SW group exhibited lower DBP values than the AM group. Although statistically significant differences were identified for SBP and DBP between some groups, the corresponding effect sizes were trivial to small (SBP: d ≈ 0.10–0.30; DBP: d < 0.25), suggesting limited clinical relevance of these differences.

Resting HR was significantly higher in sedentary individuals compared with their physically active counterparts in both sexes. Effect size analysis demonstrated a large effect in men (AM vs. SM: d ≈ 0.95; 95% CI: 4.5–9.5 bpm) and a very large effect in women (AW vs. SW: d ≈ 1.40; 95% CI: 8.0–14.0 bpm). The confidence intervals and effect sizes consistently supported the robustness of the observed associations between physical inactivity and elevated resting HR (Table 1).

Sex and an active lifestyle exerted significant influences on HRV. Regardless of physical activity level, women exhibited lower absolute values of LF-band (LF abs) compared with their male counterparts. Although LF abs showed small differences according to physical activity level, large differences were observed between sexes (AM vs. AW: d = 1.10), suggesting a strong sex-related influence. Higher absolute values of HF-band (HF abs) were observed in the physically active groups (AM and AW). Effect size analyses demonstrated large effects of physical activity in both men (d = 0.85) and women (d = 1.20), with higher HF abs values in active individuals, indicating enhanced cardiac parasympathetic modulation (Table 1).

Furthermore, an active lifestyle was associated with lower LF-band normalized units (LF nu, %) and higher HF-band normalized units (HF nu, %) in the AM and AW groups compared with their sedentary counterparts. The AW group also exhibited lower LF nu and higher HF nu values than the AM group. LF nu values were significantly higher in sedentary individuals, with large effect sizes in men (d = 0.80) and moderate-to-large effects in women (d = 0.70). Conversely, HF nu values were higher in physically active groups, also with large effect sizes. Comparisons between sexes revealed very large effect sizes for both LF nu and HF nu (d = 1.50), reinforcing the strong influence of sex on autonomic balance (Table 1).

**Figure 2:**
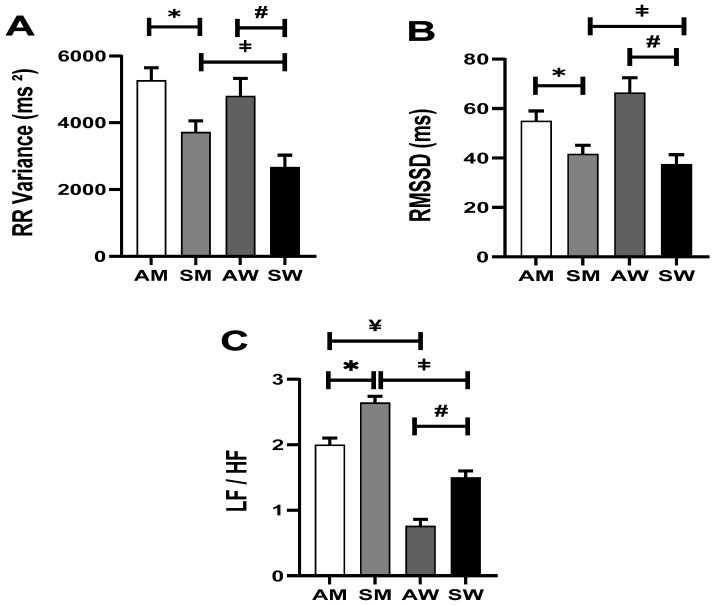
(**A**) Total RR variance; (**B**) RMSSD; (**C**) LF/HF ratio in studied groups. Values represent mean ± standard error. AM—Active Men; SM—Sedentary Men; AW—Active Women; SW—Sedentary Women; RMSSD: Root Mean Square of Successive Differences. * *p* = 0.001 AM vs. SM; # *p* = 0.013 AW vs. SW; ¥ *p* = 0.01 AM vs. AW; ‡ *p* = 0.01 SM vs. SW. Effect sizes for VAR-RR were moderate in men (d ≈ 0.75) and large in women (d ≈ 1.10), while RMSSD showed moderate-to-large effects in men (d ≈ 0.65) and large effects in women (d ≈ 1.20), indicating enhanced vagal modulation in physically active individuals. For LF/HF ratio, large effect sizes were observed between active and sedentary groups (men: d = 0.94; women: d = 1.17) and very large effects between sexes (d > 1.6), with confidence intervals described in the Section 3.

Consequently, women in all studied groups demonstrated lower LF/HF ratio, compared with men. The SM group exhibited higher LF/HF ratio values compared with the other groups (SM: 2.6 ± 0.1 vs. AM: 2.0 ± 0.1; SW: 1.5 ± 0.1; AW: 0.8 ± 0.1, *p* = 0.01). In addition, the AW group presented lower LF/HF ratio values than the AM group (Figure 2C). The LF/HF ratio was significantly higher in sedentary individuals compared with their physically active counterparts, with large effect sizes observed in men (d = 0.94, 95% CI: 0.32–0.88) and women (d = 1.17, 95% CI: 0.42–0.98). Moreover, a very large effect of sex was identified, with higher LF/HF ratio values in men than in women under both active (d = 2.03, 95% CI: 0.92–1.48) and sedentary conditions (d = 1.69, 95% CI: 0.82–1.38), suggesting a sex-related influence on cardiac autonomic modulation patterns (Figure 2C).

In terms of time-domain HRV analysis, higher total RR variance (VAR-RR) (Figure 2A) and RMSSD (Figure 2B) values were observed in the physically active groups (AM: 5272 ± 373 and AW: 4804 ± 522 ms^2^; AM: 55 ± 4 and AW: 66 ± 6 ms, respectively) compared with the sedentary groups (SM: 3724 ± 333 and SW: 2667 ± 363 ms^2^; SM: 42 ± 4 and SW: 38 ± 4 ms, *p* = 0.034 and *p* = 0.001, respectively). Furthermore, VAR-RR values were lower in the SW group (2667 ± 363 ms^2^) compared with the AM group (3724 ± 333 ms^2^; *p* = 0.01). Effect size analyses demonstrated higher VAR-RR values in active men compared with sedentary men (d = 0.75), whereas active women exhibited an even greater increase relative to sedentary women (d ≈ 1.10), suggesting a stronger association between physical activity and autonomic modulation in women. Similarly, RMSSD values demonstrated moderate-to-large effect sizes in men (d = 0.65) and large effect sizes in women (d = 1.20) (Figure 2B,C). Collectively, these findings reinforce the association between physical activity and enhanced cardiac vagal modulation.

## 4. Discussion

Given the observed relationships between HRV, physical activity, and sex, we conducted a study to examine how these factors relate to cardiac autonomic modulation. The main findings of the present study are as follows: First, women exhibited lower SBP and a more favorable LF/HF ratio compared to men. Second, participants with a sedentary lifestyle tended to show lower VAR-RR and RMSSD and higher LF/HF ratio. Notably, regular physical activity and female sex were associated with more favorable indices of cardiac autonomic modulation in the studied population. Importantly, several HRV indices and resting HR demonstrated moderate-to-very large effect sizes, reinforcing the robustness and physiological relevance of the associations between physical activity, sex, and cardiac autonomic modulation in young healthy subjects.

Basal indexes of cardiac autonomic regulation can be influenced by various factors, including sex. In the present study, it was observed that young healthy women exhibited lower SBP than young healthy men. This observation aligns with previous research, which has consistently reported that middle-aged women have the highest HRV and the lowest resting arterial pressure levels in comparison to men [22,23]. Moreover, a recent study conducted by our group demonstrated improved cardiovascular autonomic regulation and a more favorable oxidative stress profile in healthy females during the non-ovulatory phases of the estrogenic cycle, when compared to male rats and female ovariectomized rats [24]. This evidence support the notion that estrogen exerts a protective influence on the cardiovascular system through its impact on the autonomic nervous system [22,25]. In accordance with the research conducted by Hao et al., it appears that estrogen serves as a crucial mediator, acting centrally to reduce blood pressure, sympathoexcitation, and oxidative stress [26].

Importantly, low levels of physical activity are associated with adverse health outcomes. A study involving 2630 adults found that the average daily time spent in moderate-to-vigorous physical activity was less than 10 min [27]. Such insufficient levels of physical activity are directly associated with an increased risk of cardiovascular diseases and other health complications, as supported by the literature [11]. In this sense, despite the sex-related differences observed in our study, previous research has consistently shown that regular physical activity, in men or in women, can significantly enhance cardiac autonomic modulation, effectively reducing the risk of various pathophysiological conditions [14,28]. Physical activity improves HRV regardless of sex, corroborating a recent meta-analysis by El-Malahi et al. (2024) [29]. It is worth noting that the global prevalence of insufficient physical activity stands at 23.4% for men and approximately 31% for women [10]. This means that a small proportion of individuals, both men and women, are meeting the recommended guidelines of at least 150 min of moderate or 75 min of vigorous physical activity per week, as outlined by the Physical Activity Guidelines [30].

Physical inactivity has been consistently associated with a reduction in HRV, which serves as a marker for the development of CVD [28,31,32]. Our present study corroborated previously published findings [14,22] by confirming that physical inactivity is indeed linked to cardiac autonomic dysfunction. This is evident through our findings of higher sympathetic and lower parasympathetic modulation, as reflected in the LF-band and HF band, with a notable impact on the LF/HF ratio, particularly in men. Notably, the magnitude of these associations was supported by moderate-to-very large effect sizes for resting HR, LF/HF ratio, HF abs, VAR-RR, and RMSSD, suggesting that the observed differences are not only statistically significant but also physiologically meaningful.

Sex-related differences in cardiac autonomic modulation have been extensively explored in prior research. However, a recent meta-analysis has shed light on a common limitation found in many studies-inadequate control for sex-related differences [33]. As a response to this gap in the literature, our study provides innovative insights by examining HRV in the context of both sex and levels of physical activity, particularly in a cohort of healthy young adults. In this context, it is also important to note that our study population consisted predominantly of young, healthy adults with normal BMI values and preserved cardiometabolic profiles recruited through convenience sampling. Although this relative homogeneity minimized potential confounding factors and reduced interindividual variability, it may limit the generalizability of the findings to older individuals or clinical populations with established cardiometabolic dysfunction. Nevertheless, the investigation of autonomic modulation in healthy subjects may provide important insights into early physiological mechanisms involved in the development and progression of cardiovascular risk.

Our findings highlight the profound impact of an active lifestyle coupled with sex differences on HRV. Specifically, we observed significantly better HRV indices in physically active individuals compared with their sedentary counterparts. For active men, there was an impressive 41% higher values of total variance (RR interval variance) and a 34% in RMSSD in comparison to sedentary men. Similarly, physically active women exhibited significantly higher VAR-RR and RMSSD values (74% and 78%, respectively) compared with sedentary women. Additionally, physically active men exhibited a 75% lower LF/HF ratio, whereas physically active women showed a 50% lower LF/HF ratio compared with their sedentary counterparts, indicating a more favorable cardiac autonomic modulation. Furthermore, when we compared physically active men to physically active women, sex differences emerged. Physically active women displayed a 66% lower LF/HF ratio and a 20% higher RMSSD in comparison to physically active men. Our findings of higher RMSSD and lower LF/HF ratio in active women align with Seo et al. (2024), who observed greater vagal activation post-HIIT in young females [34]. These outcomes are consistent with previous research, which, despite not considering physical activity levels, demonstrated that women generally exhibit lower LF band and higher power in the HF band, indicative of parasympathetic modulation [35]. However, the interpretation of HRV spectral indices, especially LF power and the LF/HF ratio, remains controversial, since these parameters may not exclusively reflect cardiac sympathetic activity or sympathovagal balance. Although LF power has historically been interpreted as a marker of sympathetic modulation, accumulating evidence indicates that LF oscillations are strongly influenced by both sympathetic and parasympathetic components, as well as by baroreflex activity [36]. Likewise, the LF/HF ratio should be interpreted with caution, since it does not provide a direct or isolated measure of sympathovagal balance under all physiological conditions. The physiological interpretation of the LF/HF ratio as a surrogate marker of cardiac sympathovagal balance may oversimplify the complex and non-linear interactions between autonomic branches [37]. In addition, LF power cannot be considered a pure index of sympathetic cardiac control, particularly under resting conditions, where vagal modulation substantially contributes to LF oscillations [36]. Therefore, in the present study, LF/HF ratio and spectral components were interpreted in conjunction with time-domain indices, such as RMSSD and VAR-RR, which are more consistently associated with vagal modulation and overall HRV. Despite these limitations, the combined pattern of lower RMSSD and higher LF/HF ratio observed in sedentary individuals may still suggest a less favorable autonomic cardiovascular profile.

This study presents several limitations that should be considered when interpreting the findings. First, the cross-sectional design precludes the establishment of causal relationships between physical activity, sex, and HRV parameters. In addition, physical activity was assessed using the IPAQ, a subjective instrument susceptible to recall bias and potential overestimation of activity levels, while also lacking detailed characterization regarding exercise modality, intensity, and duration. Nevertheless, the IPAQ remains a widely used and validated tool in large-scale population studies. Therefore, future studies combining subjective and objective measures, such as accelerometry or wearable monitoring devices, may provide a more comprehensive assessment of habitual physical activity. Additionally, variables such as sleep quality, psychological stress, dietary habits, and actual training load were not objectively controlled and may have influenced HRV responses. Furthermore, the absence of direct hormonal measurements limits mechanistic interpretations regarding the role of estrogen in cardiac autonomic regulation. Blood pressure was measured using the auscultatory method, which, although broadly accepted in both clinical and research settings, may provide a less comprehensive assessment than ambulatory or continuous hemodynamic monitoring techniques. Furthermore, the sample size was not determined a priori. Although post hoc power analysis demonstrated high statistical power (β = 0.99) for the primary outcomes, including RR interval variance and LF/HF ratio, an a priori sample size calculation would have further strengthened the methodological rigor of the study.

Our study reaffirmed a consistent pattern in which men exhibited greater cardiac sympathetic modulation than women. Importantly, this sex-related difference was observed regardless of physical activity level in young healthy subjects. Furthermore, irrespective of sex, sedentary behavior was associated with impaired HRV parameters. Collectively, these findings suggest that female sex may be associated with a more favorable autonomic cardiovascular profile, while higher levels of physical activity are associated with improved cardiac autonomic modulation. Although the observational nature of the study precludes causal inferences, the present findings reinforce the potential relevance of physical activity as a non-pharmacological strategy associated with better autonomic cardiovascular regulation and possibly lower cardiovascular risk in both sexes. In this context, HRV analysis may represent a valuable early marker for the assessment of cardiovascular risk.

## Figures and Tables

**Table 1:** Hemodynamic and Heart rate variability parameters in active and sedentary men and women.

	AM (*n* = 37)	SM (*n* = 44)	AW (*n* = 31)	SW (*n* = 40)	Cohen’s d [95% CI]
SBP (mmHg)	117 ± 1.6	116 ± 1.4	111 ± 1.9 ¥	108 ± 1.5 ‡	AM vs. SM: 0.10 [−2.5; 4.5]
					AW vs. SW: 0.30 [0.2; 5.5]
DBP (mmHg)	73 ± 1.4	74 ± 1.2	72 ± 1.6	70 ± 1.4 ‡	All comparisons: <0.25
					[overlapping CI]
HR (bpm)	65 ± 1.2	72 ± 1.1 *	65 ± 1.4	76 ± 1.2 #‡	AM vs. SM: 0.95 [4.5; 9.5]
					AW vs. SW: 1.40 [8.0; 14.0]
LF abs (ms^2^)	1688 ± 135	1514 ± 121	891 ± 97 ¥	807 ± 132 ‡	AM vs. SM: 0.25 [−150; 500]
					AW vs. SW: 0.15 [−200; 400]
					AM vs. AW: 1.10 [500; 900]
HF abs (ms^2^)	1498 ± 205	699 ± 183 *	2114 ± 374 ¥	701 ± 200 #	AM vs. SM: 0.85 [300; 1300]
					AW vs. SW: 1.20 [900; 1800]
					AM vs. AW: 0.60 [−200; 1200]
LF nu (%)	54.5 ± 2.2	62.7 ± 2.0 *	34.7 ± 2.7 ¥	51.4 ± 2.1 #‡	AM vs. SM: 0.80 [4; 12]
					AW vs. SW: 0.70 [6; 16]
					AM vs. AW: 1.50 [15; 25]
HF nu (%)	45.4 ± 2.2	37.2 ± 2.0 *	65.2 ± 2.7 ¥	48.5 ± 2.1 #‡	AM vs. SM: 0.80 [−12; −4]
					AW vs. SW: 0.70 [−16; −6]
					AM vs. AW: 1.50 [−25; −15]

Values represent mean ± standard error. AM—Active Men; SM—Sedentary Men; AW—Active Women; SW—Sedentary Women; SBP: systolic blood pressure; DBP: diastolic blood pressure; HR: heart rate. * *p* < 0.05 AM vs. SM; # *p* = 0.01 AW vs. SW; ¥ *p* = 0.0001 AM vs. AW; ‡ *p* = 0.001 SM vs. SW. Effect sizes are presented as Cohen’s d. The 95% confidence intervals (CI) refer to the mean differences between groups (Bonferroni post hoc). Effect size magnitude was interpreted as trivial (<0.2), small (0.2–0.5), moderate (0.5–0.8), and large (>0.8).

## Data Availability

The raw data supporting the conclusions of this article will be made available by the authors on request.
